# Multilayered Composites with Modulus Gradient for Enhanced Pressure—Temperature Sensing Performance

**DOI:** 10.3390/s21144752

**Published:** 2021-07-12

**Authors:** Changyoon Jeong, Sang-Ha Hwang, Byeong-Joo Kim, Han Gi Chae, Young-Bin Park

**Affiliations:** 1 School of Mechanical Engineering, Yeungnam University, Gyeongsan 38541, Korea; 2 Hanwha Solutions, Light-Weight Composite Materials R&D Center, Advanced Materials Division, 155, Gondan-ro, Yeonseo-myeon, Sejong 30058, Korea; hwang113@hanwha.com; 3 Agency for Defense Development, Daejeon 34186, Korea; dhfldhs727@naver.com; 4 School of Materials Science and Engineering, Ulsan National Institute of Science and Technology (UNIST), UNIST-gil 50, Ulju-gun, Ulsan 44919, Korea; hgchae@unist.ac.kr; 5 Department of Mechanical and Aerospace Engineering, Ulsan National Institute of Science and Technology, Ulsan 44919, Korea; ypark@unist.ac.kr

**Keywords:** multilayered composites, modulus gradient, stress concentrating geometry, stress distribution, pressure sensing, temperature sensing

## Abstract

Highly sensitive and flexible composite sensors with pressure and temperature sensing abilities are of great importance in human motion monitoring, robotic skins, and automobile seats when checking the boarding status. Several studies have been conducted to improve the temperature-pressure sensitivity; however, they require a complex fabrication process for micro-nanostructures, which are material-dependent. Therefore, there is a need to develop the structural designs to improve the sensing abilities. Herein, we demonstrate a flexible composite with an enhanced pressure and temperature sensing performance. Its structural design consists of a multilayered composite construction with an elastic modulus gradient. Controlled stress concentration and distribution induced by a micropatterned structure between the layers improves its pressure and temperature sensing performance. The proposed composite sensor can monitor a wide range of pressure and temperature stimuli and also has potential applications as an automotive seat sensor for simultaneous human temperature detection and occupant weight sensing.

## 1. Introduction

Rapid advances in flexible pressure sensors are driving interest in electronic skin [1,2,3,4], electronic textiles [5,6,7,8,9], flexible touch displays [10,11,12,13,14,15], soft robotics [16,17], and energy harvesting [18,19,20]. Recently, flexible pressure sensors, which mimic various biostructures [1,16,21] and use a characteristic material [1,22,23,24,25,26], have attracted significant research interest. A highsensitivity, flexibility, sensing range, and durability are the major requirements for pressure sensors in various applications. In particular, pressure sensing has an applicable range, such as medium for a gentle touch (1–10 kPa) [2,27] and high for human body pressure (~100 kPa) [2,28]. Various micro-structured sensors have been suggested to enhance sensing characteristics such as sensitivity, response time, sensing range, and durability based on the mechanical and electrical properties of materials [29,30,31,32,33,34,35]. Despite their outstanding performance, these sensors are limited because they require complex and difficult-to-fabricate structures to enhance their sensing performance.

In addition, temperature is an important sensing factor that determines the credibility of biosensors for various biosignal applications and can be detected using temperature sensors [3,36,37,38] and pressure sensors [2,39]. Temperature can be an indicator of an unusual condition in the body, because a small increase in the body temperature can indicate an abnormality, such as a viral infection. Therefore, the temperature sensor must be highly sensitive to small changes in temperature. Carbon nanotubes (CNTs) [40], polyaniline [5], poly (3,4-ethylenedioxythiophene) and polystyrene sulfonate (PEDOT:PSS)/CNTs composites [41] have been used as sensor materials in resistor-type temperature sensors. Among these sensors, poly (N-iso-propyl acrylamide) (pNIPAM) is a typical temperature-responsive polymer that exhibits a coil-to-globule transition effect, inducing resistance change in composites; however, this sensing sensitivity is material-dependent and needs to escape from material dependence in order to improve its sensitivity. Therefore, the development of sensors that modify the structural design, based on mechanics, to address material-dependent sensing performance and the improvement of existing sensors remains a significant challenge.

In this study, we developed a flexible composite sensor with high pressure and temperature sensitivities owing to the multilayered structure design, which comprises an elastic modulus gradient with stress-concentrating geometries. This modulus gradient design enables the following novel strategy to improve the sensing performance (pressure and temperature). Owing to the conductive, mechanical, and temperature-responsive properties of the polymer composites consisting of pNIPAM, poly vinyl alcohol (PVA), and single-walled carbon nanotubes (SWCNTs), our multilayered composites can detect changes in temperature owing to the volume change in the sensor, in addition to the pressure changes. Each layer of the composite sensor has a different modulus value and exhibits a different deformation under varying loading conditions. The modulus gradient structure enables a simultaneous increase in sensing sensitivity under varying pressure and temperature conditions. Compared to a composite sensor consisting of a single layer or circular structure sensor [28], the proposed composite with a modulus gradient exhibits a high sensitivity of 5.77 kPa^−1^, and each layer shows different levels of deformation depending on the modulus under loading. Furthermore, unlike conventional temperature sensors that depend on the properties of the materials, the proposed multilayered composites demonstrated a temperature-sensitive response of 5.92% °C^−1^, owing to the structural design with a modulus gradient. Finally, the stress-concentrating geometry composite with an elastic modulus gradient layer resulted in stress distribution in each layer under a loading condition, which improved the sensing performance.

## 2. Materials and Methods

### 2.1. Preparation of PVA/pNIPAM/SWCNTs Composite Films

Fabricating hybrid composite and PVA (Sigma-Aldrich, Saint Louis, MO, USA), with outstanding mechanical properties, hydrolyzed to 98–99%, were used to maintain the composite shape under pressure. SWCNTs solution (KH Chemicals Co., Ltd., Daejeon, Korea), with a purity of 99% and dispersed in a water containing a dispersant, was used as the composite filler. pNIPAM (Sigma-Aldrich, Saint Louis, MO, USA), with an average molecular weight of 85,000, is a temperature-responsive material. Polymers and SWCNTs were mixed, and the polymer mixing ratio (PVA and pNIPAM) was 1:1 [37]. Subsequently, the resulting solution was poured into a micro-circular patterned wafer with a 10 μm diameter and 20 μm pitch and then demolded.

### 2.2. Fabrication of Multilayered Composites

To fabricate multilayered composites with different gradient moduli, the SWCNTs concentration was controlled by the SWCNTs’ weight. Different concentrations of composites obtained layers with different elastic moduli. The multilayered structures were constructed by stacking several layers of micro-circular structures with a thickness similar to that of a single PVA/pNIPAM/SWCNTs composite film. Two copper electrodes were attached to the top and bottom sides of the multilayered composite films using a silver paste. Thin acrylic plates positioned on the top and bottom of the electrode were used to measure the pressure, temperature, and bonding force. A composite film of 30 mm length and 5 mm width was used to measure the elastic modulus of a composite film with different SWCNTs concentrations.

### 2.3. Characterization

Microstructures of the PVA/pNIPAM/SWCNTs composites were analyzed using field-emission scanning electron microscopy (SEM; S-4800, Hitachi, Chiyoda City, Tokyo, Japan). Pressure and temperature responses of the sensors were measured using a multimeter (2002, Keithley, Cleveland, OH, USA) and an electrometer/high-resistance meter (6517B, Keithley, Cleveland, OH, USA). Electrical conductivity of the composite films was measured using a 4-point probe (CMT-SR1000N, AIT, Suwon, Korea). Elastic moduli and stress–strain curves of the composites were measured using a universal testing machine (AGX-100NX, SHIMADZU, Canby, OR, USA). Static/dynamic normal pressure was applied to the sensors using a pushing tester (Pushing machine, SnM, Korea). Heat was applied to the sensors using a hot plate (MSH-20D, DAIHAN, Korea and COAD.1006, OCEAN SCIENCE, Korea).

## 3. Results and Discussion

### 3.1. Structural Design of Multilayered Composites for Pressure Sensing Capabilities

**Figure 1:**
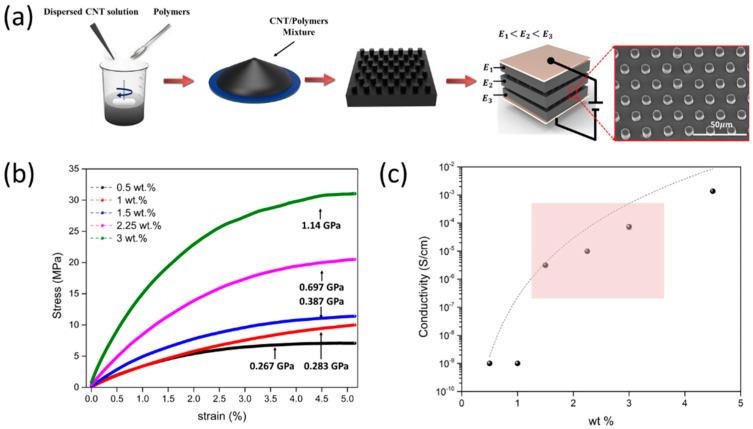
Conductive multilayer composites with modulus gradient ($E_{1}$, $E_{2}$, and $E_{3}$) and stress-concentrating geometries (**a**) Schematic of the fabrication procedure used to produce the PVA/pNIPAM/SWCNTs composite with stress-concentrating geometries (inset SEM image of the composite shows a micro-circular structure for the stress-concentrating geometries between the composite layers (scale bar: 20 μm)) (**b**) Elastic modulus is estimated by considering the stress–strain curve of composites with different CNTs wt % (**c**) Electrical conductivity with increasing wt % of CNTs (shaded box represents the optimum conductivity of the sensor design).

A schematic of the multilayered composites, with a modulus gradient for improving the sensing performance and fabrication process, is shown in Figure 1a. To fabricate the conductive and temperature-responsive composite films, PVA was dissolved in an SWCNTs solution at 70 °C for 1 h, and the resulting solution was cooled to room temperature. In addition, the polymer pNIPAM was dissolved in a mixture of SWCNTs and PVA, using a magnetic stirrer, for 48 h. This mixture was then cast onto a microhole-patterned mold (diameter 10 µm, pitch 20 µm) and dried at 30 °C for 24 h in an oven with airflow. The SEM image (Figure 1a) shows a layer with micro-circular structures for the stress-concentrating geometries under loading conditions. To design the multilayered structures with a modulus gradient, we controlled the concentration of SWCNTs in the pNIPAM/PVA polymer to fabricate the different elastic moduli. Figure 1b shows the different elastic moduli of the composite films depending on the SWCNTs concentration. The modulus of each composite film was varied by controlling the CNTs concentration (1.5 wt %, 2.25 wt %, and 3 wt %). In addition, given that the electrical conductivity of the film is an important aspect in sensor design in terms of performance, the conductivity of composite films with different CNTs concentrations was measured five times. If the CNTs concentration is too low and the resistance is high, it is difficult to distinguish between the corresponding current signal and thermal fluctuation. However, if the resistance is too low the sensor output is adversely affected by the contact resistance and sensitivity under loading conditions. The optimized conductivity for sensor design should range from 1.5 to 3 wt %, as shown in Figure 1c. The multilayered structures consisting of three SWCNTs concentration layers (1.5 wt %, 2.25 wt %, and 3 wt %) facilitated the design of the composite with a stress distribution structure for improving the sensing performance, compared to that of a single-layered composite. When the pressure applied to the multilayered composites increased from low to high, deformation initially occurred in the layer with a low modulus, followed by a deformation in the layer with a medium modulus. The layer with a high modulus exhibited final deformation under high pressure, inducing a change in electrical resistance.

The multilayered structures with modulus gradient under compressive strain can be explained using a brief equation. Force (F), which was applied to the composites with a modulus gradient, had the same value regardless of the composite layer. The modulus of each composite layer can be expressed as $E_{1}$, $E_{2}$, and $E_{3}$ $(E_{1}<E_{2}<E_{3}$). The compressive strain under pressure is summarized in Equations (1)–(3):

(1) $$\varepsilon_{1}=\frac{\sigma_{1}}{E_{1}}\;,$$

(2) $$\varepsilon_{2}=\frac{\sigma_{2}}{E_{2}}\;,$$

(3) $$\varepsilon_{3}=\frac{\sigma_{3}}{E_{3}}\;,$$

where $\sigma_{1}\;$ is the stress of the first layer composite (top layer), $\;\sigma_{2}$ is the stress of the second layer composite (middle layer), $\sigma_{3}$ is the stress of the third layer composite (bottom layer), $\varepsilon_{1}$ is the strain of the first-layer composite (top layer), $\varepsilon_{2}\;$ is the strain of the second layer composite (middle layer), and $\varepsilon_{3}$ is the strain of the third layer composite (bottom layer).

The stress of each composite layer has the same value owing to the principle of action and reaction if the area of the microstructure has the same value; therefore, the deformation of the first layer with a low modulus was large and the other layers deformed sequentially from the second to the third composite layer.

**Figure 2:**
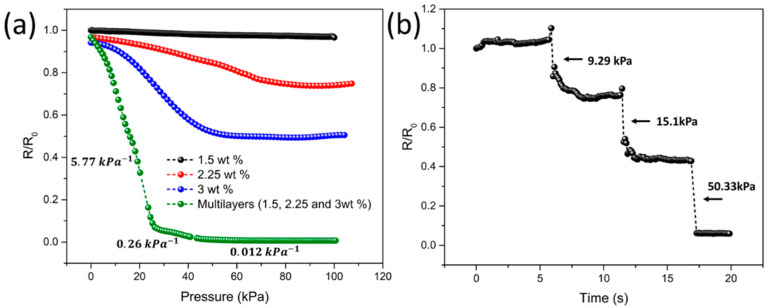
(**a**) Enhanced pressure-sensing performance of a multilayer composite with modulus gradient compared to a single-layer composite (**b**) Real-time pressure monitoring of composites for applied step loading (pressure values of 9.29 kPa, 15.1 kPa, and 50.33 kPa).

Figure 2a shows that the pressure sensitivity of multilayered composites with a modulus gradient is higher than that of a single-layered structure because of the stress distribution in each stacked layer with different elastic moduli. In particular, the pressure sensitivity and sensing range see an increase, owing to the stress distribution of each stacked layer. To investigate the real-time pressure-sensing performance of the multilayered composites, the relative resistance change was measured under incremental pressure (Figure 2b). Under a stepwise increase in pressure from 9.29 kPa to 50.33 kPa, the composite sensor showed a fast response in terms of a decreasing resistance change.

**Figure 3:**
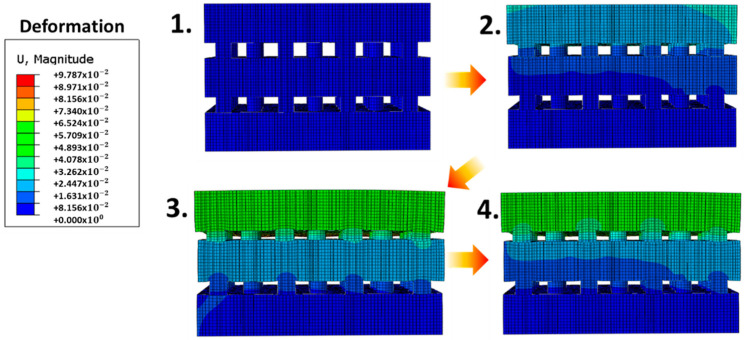
Finite-element calculations (deformation 0–9.787 cm) of the deformed multilayer composite with modulus gradient under pressure.

**Figure 4:**
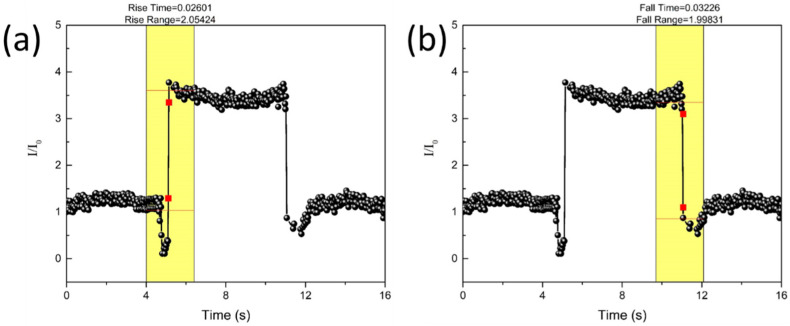
Response to loading (**a**) and unloading (**b**) of multilayer composite (low pressure of 1.6 kPa).

**Figure 5:**
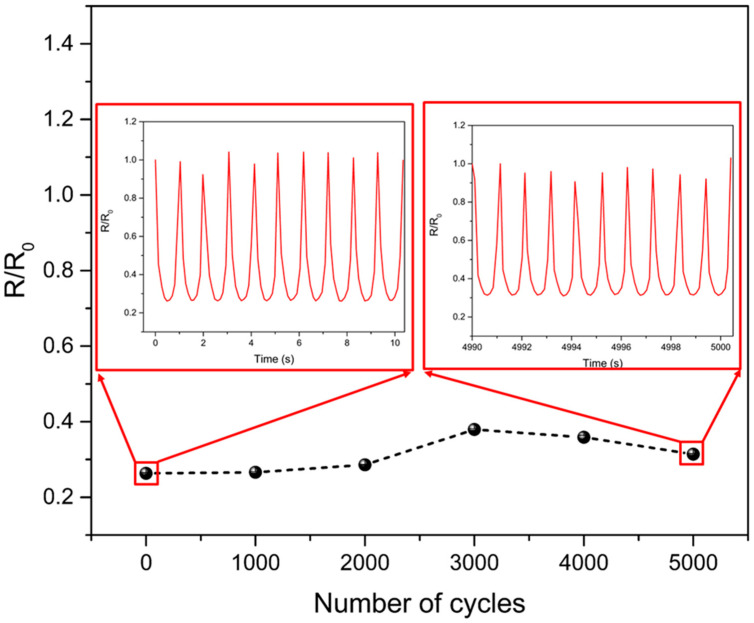
Cyclic stability test of multilayer composite under repetitive pressure (5000 cycles) of 33 kPa at a frequency of 1 Hz.

Finite-element simulations were performed using ABAQUS software (Dassault systems, Waltham, MA, USA) for structural analysis of the multilayers with a modulus gradient. The deformation in the different modulus layers was numerically calculated as a function of the applied pressure. The occupied number of the patterned circular structure and pitch ratio was approximately equal to the experimental ratio. Boundary conditions were applied to the in-plane directions, and the bottom surface was fixed. Loading conditions assigned pressure to the top surface. We used six-node linear triangular prism elements. The elastic moduli of the second and third composite layers were two and three times that of the first-layer composite, respectively (70, 140 and 210 MPa). This is similar to the proportions of the real modulus having an optimal conductivity of the composites. The Poisson’s ratio was 0.37, showing the deformation gradient under pressure. We implemented general surface-to-surface contact interaction with linear elastic deformable materials to consider the mechanical contact interaction between the stacked multilayers with a modulus gradient under the applied pressure. We then analyzed the theoretical deformation distribution. The pressure sensitivity and sensing range increased owing to the stress distribution in each stacked layer (Figure 3). In addition, multilayered stress-concentrating geometries with a modulus gradient can provide a fast response to the loading and unloading of low pressure, owing to the immediate deformation and recovery of microstructures in response to an applied pressure. At a low pressure of 1.6 kPa, the proposed composite sensor demonstrated fast response and relaxation times of 26 ms and 32 ms, respectively (Figure 4). Furthermore, during the repetitive cycles of pressure loads of 33 kPa at a frequency of 1 Hz, the sensor exhibited a reliable and uniform pressure sensing performance up to 5000 cycles (Figure 5).

### 3.2. Structural Design of Multilayered Composites for Temperature Sensing Capabilities

**Figure 6:**
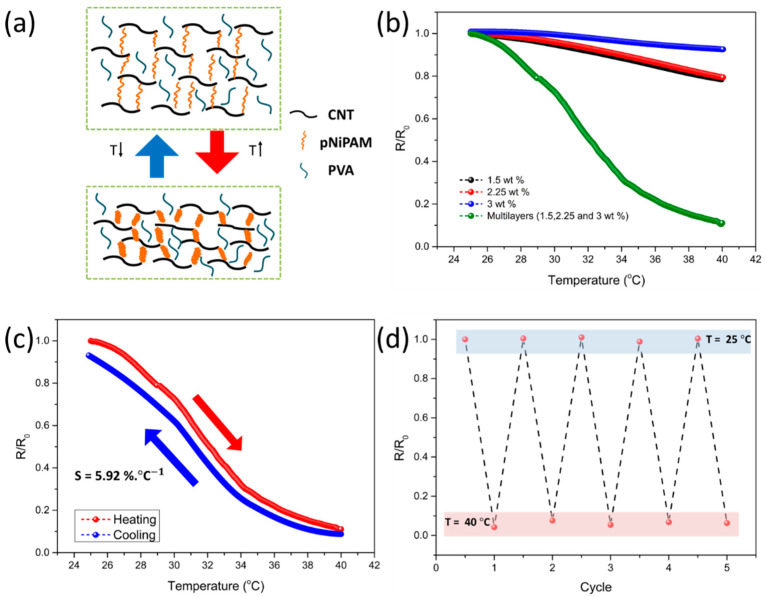
Enhanced temperature-sensing performances of a multilayer sensor with modulus gradient compared to that of a single-layer sensor (**a**) Schematic illustration of the sensing mechanism of PVA/pNIPAM/SWCNTs composites with a change in temperature (**b**) Relative resistance of multilayer sensor with modulus gradient and single-layer sensor in response to a change in temperature (**c**) Relative resistance changes based on temperature, with increments/decrements rates of 0.43 °C/s and 0.012 °C/s (**d**) Repetitive measurements of the relative resistance changes over five cycles between 25 °C and 40 °C.

In addition to improving the sensing performance under an applied pressure owing to the structural design, the temperature-sensing ability can also be improved by exploiting the structural design of the composites. To effectively detect the temperature changes and measure the resistance change in the composite films, several factors must be considered. The chain structure of pNIPAM is initially transformed into a coiled structure with an increase in temperature, which decreases the volume of the composite [21,42,43,44]. The decrease in volume results in a dense percolation conductive network, which decreases the relative resistance, as illustrated in Figure 6a. Additionally, it is known that the thermal coefficients of individual components of the sensing materials affect the temperature-dependent change in resistance [45,46,47]. In addition, there seems to be an electron-hopping effect between the interfaces of the CNTs in the pNIPAM/PVA/SWCNTs composite. Electron transport increases with an increase in temperature, owing to activated electron hopping [48]. To structurally reinforce this material-temperature response mechanism, we compared the thermal sensitivity of the multilayered composites to that of a single-layer composite, as shown in Figure 6b. The former exhibits a higher sensitivity than the latter, owing to the deformation gradient effect under temperature changes. With an increase in temperature from 25 °C to 40 °C, our multilayered composite sensor was used to monitor the temperature changes, and the results revealed a stable operation (Figure 6c). The sensitivity was estimated to be 5.92% °C^−1^ of the relative electrical resistance (R/R_0_, where R is the current resistance and R_0_ is the initial resistance), which were obtained by increasing the temperature from 25 °C to 40 °C. A higher sensitivity was achieved owing to the multilayered structural effect, which increased the temperature-sensing sensitivity. In Figure 6d, repeated changes in temperature in the range of 25–40 °C resulted in changes in resistance, confirming the reproducibility and consistency of the temperature measurements.

### 3.3. Multilayered Composites for Simultaneous Pressure-Temperature Sensing Capabilities

**Figure 7:**
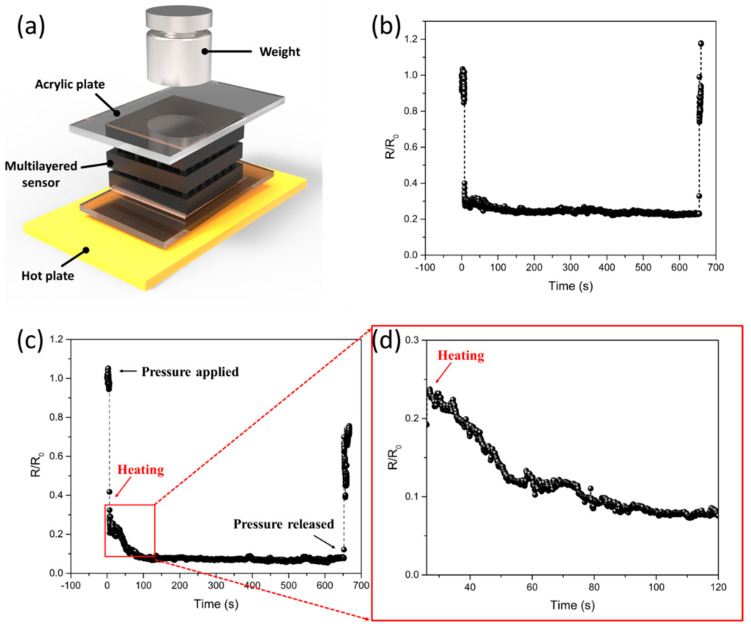
Piezoresistive pressure-temperature sensing performances of multilayer sensor with modulus gradient (**a**) Schematic of the multilayer sensor for detecting pressure and temperature changes (**b**) Real-time monitoring of pressure (mass 100 g) by multilayer sensor (**c**) Real-time monitoring of relative resistance changes under pressure (100 g), and (**d**) expanded signal showing the temperature response of the multilayer sensor (25–40 °C).

To explore the suitability of multilayered composites for pressure and temperature sensing applications, we placed the composites on a hotplate and applied pressure by adding mass (Figure 7a). A mass of 100 g was placed on the composites, resulting in a decrease in the relative resistance (Figure 7b). When the pressure was maintained and subsequently released, the relative resistance assumed its original value. Figure 7c shows the pressure and temperature responses of the multilayered composites with a modulus gradient. Pressure was applied to the multilayered composites, which were then heated to 40 °C. The expanded signal showed a decrease in the relative resistance as the temperature increased (Figure 7d). The temperature was maintained at 40 °C. When the mass was removed after 500 s at a certain temperature, the relative resistance returned to its original value, where the resistance decreased with temperature.

**Figure 8:**
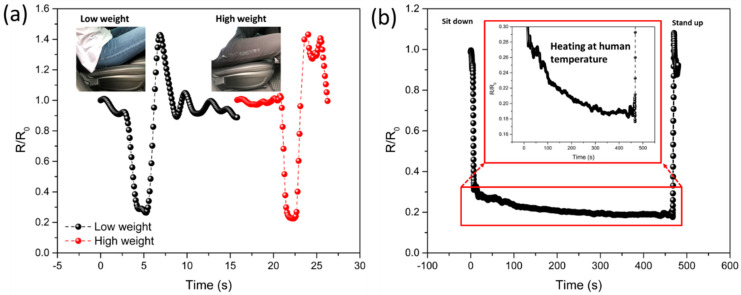
Application of pressure-temperature sensing abilities of multilayer sensor with modulus gradient (**a**) Real-time monitoring of low and high human mass signals getting in and out of the car seat (inset image shows results for a low mass (60 kg) and a high mass (85 kg) (**b**) Monitored real-time signals of sitting down on the seat detected by a multilayer sensor and showing heat transfer from the human body to the multilayer sensor.

Multilayered composites with a high sensitivity and large pressure-sensing range could be used for applications that require large pressure measurements, such as automotive seats. To detect the presence or absence of an occupant, a sensor was attached to the car seat. The composite sensor could detect the pressure applied by the occupant and differentiate high (85 kg) and low (60 kg) masses (Figure 8a). In addition, when the occupant got out of the seat, it could detect the sweeping movement of the individual’s posterior. In particular, to detect the situation in which an individual enters or exits a vehicle accurately, it is important to monitor the change in temperature in addition to the change in pressure. Figure 8b shows the electric signal obtained from an occupant seated in a car. Initially, when the individual sits down, the relative resistance decreases owing to the applied pressure.

As the multilayered composite is heated by the human body, it responds and induces an additional decrease in the relative resistance. Therefore, it can be applied to monitor the exit or entry in vehicles based on the relative resistance change associated with temperature and pressure.

## 4. Conclusions

In this study, we demonstrated a flexible composite sensor with an increased sensing ability under varying pressure and temperature changes, owing to the structural design of composites. The developed composite sensor has a structural design consisting of multilayered PVA/pNIPAM/SWCNTs composite layers with a modulus gradient and controlled stress-concentrating structures. This structure can improve the sensing performance compared to a single-layered composite, owing to the structural design for stress distribution. It exhibited high pressure (5.77 kPa^−1^) and temperature (5.92% °C^−1^) sensitivities. Simulations confirmed that the increase in sensing capabilities under varying pressure and temperature conditions owed to the effective stress-concentration geometry and stress distribution between the modulus-gradient layers. In particular, it was observed that temperature sensing sensitivity increased because of the multilayered structural effect, induced by the modulus gradient in addition to the material characteristics. These sensing abilities enabled our sensor to monitor the human body temperature and pressure to accurately detect human movements. The suggested multilayered composites could be useful for improving the sensing performance of existing sensors with limited performance.

## Data Availability

Data is contained within the article.
